# (*S*)-4-*tert*-Butyl-2-(1,2,3,4-tetra­hydro­isoquinolin-3-yl)-1,3-thia­zole

**DOI:** 10.1107/S160053681203173X

**Published:** 2012-07-18

**Authors:** Sunayna Pawar, Thavendran Govender, Hendrik G. Kruger, Glenn E. M. Maguire

**Affiliations:** a School of Pharmacy and Pharmacology, University of KwaZulu-Natal, Durban 4000, South Africa; b School of Chemistry, University of KwaZulu-Natal, Durban 4000, South Africa

## Abstract

In the title compound, C_16_H_20_N_2_S, a potential tetra­hydro­isoquinoline (TIQ) thia­zole ligand, the N-containing six-membered ring of the TIQ unit adopts a half-chair conformation. There are four mol­ecules in the asymmetric unit. No classical hydrogen bonds or π–π inter­actions were found in the crystal structure.

## Related literature

For reactions associated with tetra­hydro­isoquinoline ligands, see: Chakka *et al.* (2010 ▶); Naicker *et al.* (2010 ▶); Kawthekar *et al.* (2010 ▶); Peters *et al.* (2010 ▶); Pawar *et al.* (2012 ▶). For related structures, see: Aubry *et al.* (2006 ▶); Naicker *et al.* (2011*a* ▶,*b* ▶); Pawar *et al.* (2011 ▶).
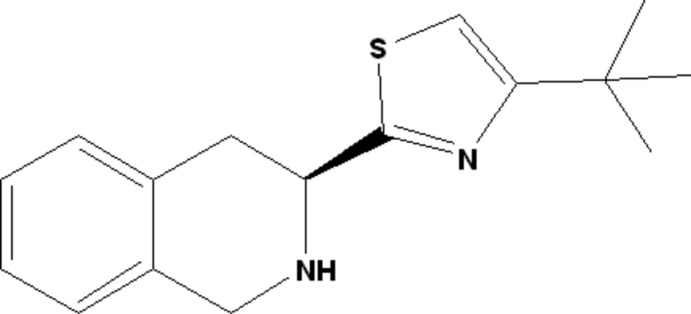


## Experimental

### 

#### Crystal data

C_16_H_20_N_2_S
*M* *_r_* = 272.40
Monoclinic, 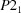

*a* = 10.0534 (9) Å
*b* = 13.0076 (12) Å
*c* = 23.808 (2) Å
β = 102.076 (1)°
*V* = 3044.5 (5) Å^3^
*Z* = 8
Mo *K*α radiation
μ = 0.20 mm^−1^
*T* = 173 K
0.18 × 0.16 × 0.08 mm

#### Data collection

Bruker Kappa DUO APEXII diffractometer
Absorption correction: multi-scan (*SADABS*; Bruker, 2006 ▶) *T*_min_ = 0.965, *T*_max_ = 0.984
39811 measured reflections
15130 independent reflections
12021 reflections with *I* > 2σ(*I*)
*R*_int_ = 0.035
Standard reflections: ?

#### Refinement

*R*[*F* ^2^ > 2σ(*F* ^2^)] = 0.092
*wR*(*F* ^2^) = 0.299
*S* = 1.04
15130 reflections
710 parameters
5 restraints
H atoms treated by a mixture of independent and constrained refinement
Δρ_max_ = 0.99 e Å^−3^
Δρ_min_ = −0.76 e Å^−3^
Absolute structure: Flack (1983 ▶), 7172 Friedel pairs
Flack parameter: 0.04 (13)

### 

Data collection: *SAINT* (Bruker, 2006 ▶); cell refinement: *SAINT*; data reduction: *SAINT*; program(s) used to solve structure: *SHELXS97* (Sheldrick, 2008 ▶); program(s) used to refine structure: *SHELXL97* (Sheldrick, 2008 ▶); molecular graphics: *OLEX2* (Dolomanov *et al.*, 2009 ▶); software used to prepare material for publication: *SHELXL97*.

## Supplementary Material

Crystal structure: contains datablock(s) I, global. DOI: 10.1107/S160053681203173X/gw2121sup1.cif

Structure factors: contains datablock(s) I. DOI: 10.1107/S160053681203173X/gw2121Isup2.hkl

Supplementary material file. DOI: 10.1107/S160053681203173X/gw2121Isup3.cml

Additional supplementary materials:  crystallographic information; 3D view; checkCIF report

## Figures and Tables

**Fig. 1.**
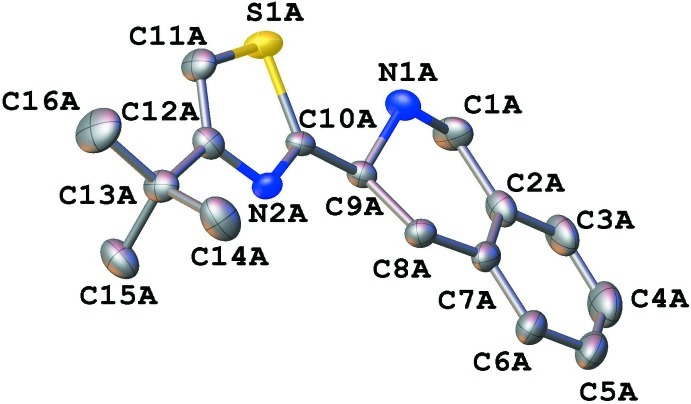
The crystal structure of the title compound with atomic numbering. Displacement ellipsoids are drawn at 50% probability.

## supplementary crystallographic information

### Comment

We have recently reported a range of tetrahydroisoquinoline (TIQ) structures that have been employed as ligands for catalysis of a number of reactions, Chakka *et al.* 2010, Naicker *et al.*, 2010, Kawthekar *et al.*, 2010 and Peters *et al.*, 2010. The title compound is from a new family of TIQ thiazole derivatives that we have tested for catalytic activity in the Henry reaction, (Pawar *et al.*, 2012).

The absolute stereochemistry was confirmed to be *S* at the C9 position from two-dimensional NMR spectroscopy experiments (Aubry *et al.*, 2006), (Fig. 1). From the crystal structure it is evident that the *N*-containing six membered ring assumes a half chair conformation [Q = 0.493 (6) Å, θ = 49.5 (7)° and φ = 322.8 (9)°]. This is similar to our previously reported structures which also assume this conformation (Naicker *et al.*, 2011*a*,*b* and Pawar *et al.* 2011). The torsion angle for C1A—N1A—C9A—C10A is -170.1 (5)°; C1B—N1B—C9B—C10B is -173.4 (5)°; C1C—N1C—C9C—C10C is -170.5 (5)° and C1D—N1D—C9D—C10D is -169.1 (5)°. For the plain formed by the atoms C1—C2—C7—C8—C9—N1 the maximum displacement from planarity for N1A is 0.446 Å and for C9A 0.299 Å; N1B is 0.384 Å and for C9B 0.296 Å; N1C is 0.427 Å and for C9C 0.258 Å and N1D is 0.286 Å and for C9D 0.473 Å. There are no hydrogen bonding or π–π interactions in the crystal lattice.

### Experimental

The *N*-protected thiazole (3 mmol) was dissolved in THF (15 ml), to this 12 *M* HCl (15 ml) was added slowly and the reaction mixture was stirred at room temperature for 2 h. The reaction was monitored by TLC using EtOAc/Hexane (20:80, *R*_f_ = 0.5). After this time the THF was evaporated under vacuum. Aqueous saturated NaHCO_3_ solution, was then slowly poured into the mixture which was then extracted with CH_2_Cl_2_ (3 *x* 30 ml). The combined organic layers waere dried over MgSO_4_. The solvent was then evaporated under reduced pressure. The residue was purified by column chromatography on silica gel (deactivated with 5% Et_3_N) with Et_3_N/EtOAc/Hexane (5/8/100) as the eluent to afford the TIQ thiazole as a yellow solid (0.27 g, yield 95%).

Recrystallization from mixture of hexane and dichloromethane at room temperature afforded colourless crystals suitable for X-ray analysis.

### Refinement

The crystal was twinned. When the structure was attempted to be refined with a P21/c space group the *R* factor rose to 20%. All hydrogen atoms, except H1A, H1B, H1C and H1D, were positioned geometrically with C—H distances ranging from 0.95 Å to 0.99 Å and refined as riding on their parent atoms with *U*_iso_ (H) = 1.2 - 1.5 *U*_eq_ (C). The positions of H1A, H1B, H1C and H1D were located in difference electron density maps and refined with bond length constraints [*d*(N—H) = 0.97 (2) Å] and *U*_iso_ = 1.5 *U*_eq_ (N).

### Crystal data

**Table:**

C_16_H_20_N_2_S	*F*(000) = 1168
*M**_r_* = 272.40	*D*_x_ = 1.189 Mg m^−^^3^
Monoclinic, *P*2_1_	Mo *K*α radiation, λ = 0.71073 Å
*a* = 10.0534 (9) Å	Cell parameters from 39811 reflections
*b* = 13.0076 (12) Å	θ = 1.6–28.4°
*c* = 23.808 (2) Å	µ = 0.20 mm^−^^1^
β = 102.076 (1)°	*T* = 173 K
*V* = 3044.5 (5) Å^3^	Block, colourless
*Z* = 8	0.18 × 0.16 × 0.08 mm

### Data collection

**Table:**

Bruker Kappa DUO APEXII diffractometer	15130 independent reflections
Radiation source: fine-focus sealed tube	12021 reflections with *I* > 2σ(*I*)
Graphite monochromator	*R*_int_ = 0.035
0.5° φ scans and ω scans	θ_max_ = 28.4°, θ_min_ = 1.6°
Absorption correction: multi-scan (*SADABS*; Bruker, 2006)	*h* = −13→13
*T*_min_ = 0.965, *T*_max_ = 0.984	*k* = −17→17
39811 measured reflections	*l* = −31→31

### Refinement

**Table:**

Refinement on *F*^2^	Secondary atom site location: difference Fourier map
Least-squares matrix: full	Hydrogen site location: inferred from neighbouring sites
*R*[*F*^2^ > 2σ(*F*^2^)] = 0.092	H atoms treated by a mixture of independent and constrained refinement
*wR*(*F*^2^) = 0.299	*w* = 1/[σ^2^(*F*_o_^2^) + (0.193*P*)^2^ + 3.4647*P*] where *P* = (*F*_o_^2^ + 2*F*_c_^2^)/3
*S* = 1.04	(Δ/σ)_max_ = 0.001
15130 reflections	Δρ_max_ = 0.99 e Å^−^^3^
710 parameters	Δρ_min_ = −0.76 e Å^−^^3^
5 restraints	Absolute structure: Flack (1983), 7172 Friedel pairs
Primary atom site location: structure-invariant direct methods	Flack parameter: 0.04 (13)

### Special details

**Table:**

Experimental. Half sphere of data collected using the Bruker *SAINT* software package. Crystal to detector distance = 30 mm; combination of φ and ω scans of 0.5°, 30 s per °, 2 iterations.
Geometry. All e.s.d.'s (except the e.s.d. in the dihedral angle between two l.s. planes) are estimated using the full covariance matrix. The cell e.s.d.'s are taken into account individually in the estimation of e.s.d.'s in distances, angles and torsion angles; correlations between e.s.d.'s in cell parameters are only used when they are defined by crystal symmetry. An approximate (isotropic) treatment of cell e.s.d.'s is used for estimating e.s.d.'s involving l.s. planes.
Refinement. Refinement of *F*^2^ against ALL reflections. The weighted *R*-factor *wR* and goodness of fit *S* are based on *F*^2^, conventional *R*-factors *R* are based on *F*, with *F* set to zero for negative *F*^2^. The threshold expression of *F*^2^ > σ(*F*^2^) is used only for calculating *R*-factors(gt) *etc*. and is not relevant to the choice of reflections for refinement. *R*-factors based on *F*^2^ are statistically about twice as large as those based on *F*, and *R*- factors based on ALL data will be even larger.

### Fractional atomic coordinates and isotropic or equivalent isotropic displacement parameters (Å^2^)

**Table:**

	*x*	*y*	*z*	*U*_iso_*/*U*_eq_	
S1A	0.09853 (14)	0.61309 (11)	0.11586 (7)	0.0419 (3)	
N1A	0.3267 (5)	0.6150 (4)	0.2152 (2)	0.0378 (10)	
H1A	0.264 (6)	0.604 (7)	0.240 (3)	0.057*	
N2A	0.1638 (5)	0.4234 (3)	0.11006 (19)	0.0329 (9)	
C1A	0.4608 (7)	0.6376 (5)	0.2515 (3)	0.0489 (15)	
H1A1	0.4494	0.6893	0.2806	0.059*	
H1A2	0.5193	0.6683	0.2273	0.059*	
C2A	0.5322 (6)	0.5450 (5)	0.2818 (2)	0.0422 (13)	
C3A	0.6510 (7)	0.5576 (7)	0.3254 (3)	0.0562 (18)	
H3A	0.6837	0.6248	0.3361	0.067*	
C4A	0.7189 (8)	0.4742 (8)	0.3521 (3)	0.068 (2)	
H4A	0.7985	0.4846	0.3810	0.082*	
C5A	0.6754 (7)	0.3759 (7)	0.3382 (3)	0.0571 (18)	
H5A	0.7231	0.3191	0.3580	0.068*	
C6A	0.5607 (6)	0.3594 (5)	0.2949 (3)	0.0449 (14)	
H6A	0.5309	0.2914	0.2845	0.054*	
C7A	0.4889 (6)	0.4442 (5)	0.2665 (2)	0.0367 (11)	
C8A	0.3660 (5)	0.4250 (4)	0.2192 (2)	0.0333 (10)	
H8A1	0.2868	0.4079	0.2360	0.040*	
H8A2	0.3837	0.3661	0.1954	0.040*	
C9A	0.3349 (5)	0.5207 (4)	0.1816 (2)	0.0280 (9)	
H9A	0.4107	0.5299	0.1608	0.034*	
C10A	0.2059 (5)	0.5086 (4)	0.1376 (2)	0.0278 (9)	
C11A	−0.0054 (6)	0.5346 (5)	0.0667 (3)	0.0424 (13)	
H11A	−0.0869	0.5561	0.0415	0.051*	
C12A	0.0464 (5)	0.4374 (4)	0.0691 (2)	0.0351 (11)	
C13A	−0.0077 (5)	0.3479 (5)	0.0315 (3)	0.0403 (12)	
C14A	−0.0372 (8)	0.2563 (6)	0.0695 (3)	0.0591 (18)	
H14A	0.0414	0.2457	0.1011	0.089*	
H14B	−0.0538	0.1938	0.0461	0.089*	
H14C	−0.1176	0.2721	0.0852	0.089*	
C15A	0.1035 (7)	0.3142 (6)	0.0002 (3)	0.0547 (17)	
H15A	0.1256	0.3713	−0.0231	0.082*	
H15B	0.0710	0.2555	−0.0248	0.082*	
H15C	0.1850	0.2941	0.0284	0.082*	
C16A	−0.1361 (8)	0.3772 (8)	−0.0118 (4)	0.072 (2)	
H16A	−0.2015	0.4085	0.0084	0.107*	
H16B	−0.1762	0.3156	−0.0322	0.107*	
H16C	−0.1132	0.4266	−0.0394	0.107*	
S1B	0.60664 (15)	0.34925 (11)	0.11989 (6)	0.0400 (3)	
N1B	0.8906 (6)	0.3639 (4)	0.2019 (2)	0.0462 (12)	
H1B	0.940 (7)	0.380 (7)	0.171 (3)	0.069*	
N2B	0.6586 (5)	0.5416 (4)	0.1216 (2)	0.0374 (10)	
C1B	0.9826 (6)	0.3481 (5)	0.2572 (3)	0.0439 (13)	
H1B1	1.0552	0.3000	0.2519	0.053*	
H1B2	0.9313	0.3148	0.2835	0.053*	
C2B	1.0472 (6)	0.4433 (5)	0.2851 (2)	0.0364 (11)	
C3B	1.1645 (7)	0.4370 (6)	0.3293 (3)	0.0519 (16)	
H3B	1.2013	0.3716	0.3418	0.062*	
C4B	1.2274 (7)	0.5265 (8)	0.3551 (3)	0.062 (2)	
H4B	1.3074	0.5220	0.3844	0.074*	
C5B	1.1729 (8)	0.6196 (7)	0.3377 (3)	0.0611 (19)	
H5B	1.2160	0.6799	0.3553	0.073*	
C6B	1.0559 (7)	0.6296 (5)	0.2950 (3)	0.0478 (14)	
H6B	1.0185	0.6955	0.2841	0.057*	
C7B	0.9939 (5)	0.5401 (4)	0.2681 (2)	0.0343 (11)	
C8B	0.8711 (6)	0.5515 (4)	0.2204 (2)	0.0374 (11)	
H8B1	0.8994	0.5803	0.1862	0.045*	
H8B2	0.8071	0.6006	0.2323	0.045*	
C9B	0.7984 (6)	0.4497 (4)	0.2043 (3)	0.0390 (12)	
H9B	0.7481	0.4333	0.2352	0.047*	
C10B	0.6936 (5)	0.4573 (4)	0.1491 (2)	0.0346 (11)	
C11B	0.5115 (6)	0.4238 (5)	0.0688 (3)	0.0420 (12)	
H11B	0.4397	0.3987	0.0395	0.050*	
C12B	0.5500 (5)	0.5239 (4)	0.0745 (2)	0.0313 (10)	
C13B	0.4973 (5)	0.6145 (5)	0.0360 (3)	0.0401 (12)	
C14B	0.6112 (7)	0.6504 (6)	0.0065 (3)	0.0520 (17)	
H14D	0.5775	0.7064	−0.0203	0.078*	
H14E	0.6402	0.5929	−0.0147	0.078*	
H14F	0.6886	0.6748	0.0355	0.078*	
C15B	0.3705 (7)	0.5821 (7)	−0.0092 (4)	0.064 (2)	
H15D	0.2956	0.5670	0.0101	0.097*	
H15E	0.3917	0.5207	−0.0295	0.097*	
H15F	0.3439	0.6382	−0.0367	0.097*	
C16B	0.4602 (7)	0.7032 (5)	0.0722 (3)	0.0516 (16)	
H16D	0.4428	0.7654	0.0485	0.077*	
H16E	0.5356	0.7158	0.1049	0.077*	
H16F	0.3783	0.6852	0.0863	0.077*	
S1C	0.49001 (18)	1.13218 (11)	0.37976 (7)	0.0457 (4)	
N1C	0.6940 (6)	1.1111 (4)	0.2993 (2)	0.0506 (13)	
H1C	0.746 (8)	1.109 (8)	0.3381 (15)	0.076*	
N2C	0.5430 (5)	0.9411 (4)	0.3832 (2)	0.0380 (10)	
C1C	0.7239 (7)	1.1284 (5)	0.2438 (3)	0.0494 (15)	
H1C1	0.7998	1.1783	0.2479	0.059*	
H1C2	0.6434	1.1604	0.2188	0.059*	
C2C	0.7620 (6)	1.0332 (5)	0.2141 (3)	0.0415 (12)	
C3C	0.8347 (7)	1.0409 (8)	0.1699 (3)	0.060 (2)	
H3C	0.8608	1.1064	0.1582	0.072*	
C4C	0.8679 (8)	0.9533 (8)	0.1435 (3)	0.066 (2)	
H4C	0.9169	0.9583	0.1135	0.079*	
C5C	0.8294 (8)	0.8560 (8)	0.1607 (3)	0.064 (2)	
H5C	0.8532	0.7956	0.1427	0.077*	
C6C	0.7578 (7)	0.8487 (6)	0.2034 (3)	0.0528 (15)	
H6C	0.7305	0.7830	0.2144	0.063*	
C7C	0.7244 (6)	0.9369 (5)	0.2312 (2)	0.0385 (12)	
C8C	0.6495 (6)	0.9268 (5)	0.2803 (2)	0.0382 (12)	
H8C1	0.5715	0.8795	0.2686	0.046*	
H8C2	0.7116	0.8963	0.3140	0.046*	
C9C	0.5986 (6)	1.0286 (5)	0.2972 (3)	0.0412 (12)	
H9C	0.5175	1.0469	0.2667	0.049*	
C10C	0.5504 (5)	1.0230 (4)	0.3527 (3)	0.0366 (11)	
C11C	0.4493 (6)	1.0600 (5)	0.4341 (3)	0.0406 (12)	
H11C	0.4064	1.0862	0.4631	0.049*	
C12C	0.4867 (5)	0.9611 (4)	0.4301 (2)	0.0344 (11)	
C13C	0.4733 (6)	0.8751 (5)	0.4722 (3)	0.0422 (13)	
C14C	0.4029 (8)	0.7853 (6)	0.4391 (4)	0.0607 (19)	
H14G	0.3969	0.7287	0.4656	0.091*	
H14H	0.4548	0.7629	0.4108	0.091*	
H14I	0.3112	0.8057	0.4194	0.091*	
C15C	0.6172 (7)	0.8470 (6)	0.5036 (3)	0.0574 (18)	
H15G	0.6126	0.7973	0.5341	0.086*	
H15H	0.6646	0.9090	0.5204	0.086*	
H15I	0.6666	0.8165	0.4763	0.086*	
C16C	0.3907 (9)	0.9113 (7)	0.5163 (3)	0.065 (2)	
H16G	0.3037	0.9399	0.4960	0.098*	
H16H	0.4421	0.9642	0.5410	0.098*	
H16I	0.3739	0.8528	0.5398	0.098*	
S1D	0.98789 (17)	0.88108 (13)	0.38079 (7)	0.0479 (4)	
N1D	1.1117 (5)	0.8850 (4)	0.2825 (2)	0.0367 (9)	
H1D	1.023 (4)	0.882 (6)	0.257 (2)	0.055*	
N2D	1.0684 (5)	1.0679 (4)	0.3918 (2)	0.0388 (10)	
C1D	1.2058 (7)	0.8614 (5)	0.2455 (3)	0.0449 (14)	
H1D1	1.1619	0.8113	0.2161	0.054*	
H1D2	1.2871	0.8277	0.2690	0.054*	
C2D	1.2522 (5)	0.9536 (4)	0.2152 (2)	0.0353 (11)	
C3D	1.3220 (7)	0.9387 (6)	0.1714 (3)	0.0480 (14)	
H3D	1.3418	0.8708	0.1609	0.058*	
C4D	1.3641 (8)	1.0233 (7)	0.1421 (3)	0.0600 (19)	
H4D	1.4127	1.0127	0.1124	0.072*	
C5D	1.3343 (7)	1.1189 (7)	0.1569 (3)	0.0601 (19)	
H5D	1.3612	1.1762	0.1371	0.072*	
C6D	1.2647 (6)	1.1354 (5)	0.2008 (3)	0.0419 (12)	
H6D	1.2439	1.2035	0.2105	0.050*	
C7D	1.2251 (5)	1.0524 (4)	0.2309 (2)	0.0329 (10)	
C8D	1.1525 (5)	1.0723 (4)	0.2792 (2)	0.0319 (10)	
H8D1	1.0565	1.0908	0.2632	0.038*	
H8D2	1.1961	1.1305	0.3029	0.038*	
C9D	1.1581 (5)	0.9752 (4)	0.3170 (2)	0.0303 (10)	
H9D	1.2551	0.9635	0.3366	0.036*	
C10D	1.0758 (5)	0.9858 (4)	0.3624 (2)	0.0323 (10)	
C11D	0.9375 (6)	0.9549 (6)	0.4329 (3)	0.0507 (16)	
H11D	0.8815	0.9317	0.4579	0.061*	
C12D	0.9900 (6)	1.0518 (5)	0.4328 (2)	0.0390 (12)	
C13D	0.9748 (6)	1.1356 (6)	0.4738 (2)	0.0447 (14)	
C14D	0.9180 (17)	1.2287 (8)	0.4417 (4)	0.124 (6)	
H14J	0.8767	1.2729	0.4667	0.186*	
H14K	0.8488	1.2085	0.4081	0.186*	
H14L	0.9911	1.2663	0.4291	0.186*	
C15D	1.1122 (8)	1.1564 (7)	0.5130 (4)	0.065 (2)	
H15J	1.1432	1.0946	0.5354	0.098*	
H15K	1.1036	1.2132	0.5390	0.098*	
H15L	1.1783	1.1748	0.4897	0.098*	
C16D	0.8815 (11)	1.1057 (10)	0.5137 (5)	0.094 (4)	
H16J	0.7938	1.0826	0.4910	0.141*	
H16K	0.8671	1.1654	0.5369	0.141*	
H16L	0.9232	1.0500	0.5391	0.141*	

### Atomic displacement parameters (Å^2^)

**Table:**

	*U*^11^	*U*^22^	*U*^33^	*U*^12^	*U*^13^	*U*^23^
S1A	0.0386 (7)	0.0278 (6)	0.0569 (8)	0.0050 (5)	0.0043 (6)	−0.0021 (6)
N1A	0.037 (2)	0.029 (2)	0.045 (2)	−0.0051 (18)	0.0034 (18)	−0.012 (2)
N2A	0.039 (2)	0.0227 (19)	0.034 (2)	0.0043 (16)	0.0005 (17)	−0.0028 (16)
C1A	0.048 (3)	0.033 (3)	0.059 (4)	−0.004 (2)	−0.003 (3)	−0.016 (3)
C2A	0.033 (3)	0.056 (4)	0.035 (3)	−0.007 (2)	0.002 (2)	−0.015 (3)
C3A	0.048 (3)	0.076 (5)	0.040 (3)	−0.003 (3)	−0.002 (3)	−0.023 (3)
C4A	0.055 (4)	0.099 (7)	0.043 (4)	0.001 (4)	−0.005 (3)	−0.012 (4)
C5A	0.051 (4)	0.076 (5)	0.041 (3)	0.015 (4)	0.002 (3)	0.009 (3)
C6A	0.046 (3)	0.050 (4)	0.038 (3)	0.006 (3)	0.005 (2)	0.005 (3)
C7A	0.037 (3)	0.041 (3)	0.033 (3)	0.005 (2)	0.011 (2)	0.006 (2)
C8A	0.032 (2)	0.030 (2)	0.038 (3)	−0.0001 (19)	0.006 (2)	−0.001 (2)
C9A	0.027 (2)	0.024 (2)	0.034 (2)	−0.0040 (16)	0.0069 (18)	−0.0049 (18)
C10A	0.026 (2)	0.025 (2)	0.033 (2)	0.0010 (17)	0.0089 (18)	−0.0025 (18)
C11A	0.030 (3)	0.045 (3)	0.045 (3)	0.000 (2)	−0.007 (2)	−0.007 (3)
C12A	0.032 (2)	0.037 (3)	0.038 (3)	−0.007 (2)	0.011 (2)	−0.005 (2)
C13A	0.029 (2)	0.041 (3)	0.046 (3)	−0.003 (2)	−0.003 (2)	−0.009 (3)
C14A	0.066 (4)	0.055 (4)	0.060 (4)	−0.020 (3)	0.022 (3)	−0.013 (3)
C15A	0.047 (3)	0.068 (5)	0.051 (4)	−0.008 (3)	0.015 (3)	−0.021 (3)
C16A	0.047 (4)	0.076 (6)	0.076 (5)	−0.005 (4)	−0.022 (4)	−0.010 (4)
S1B	0.0400 (7)	0.0309 (6)	0.0465 (8)	−0.0035 (5)	0.0027 (6)	0.0004 (6)
N1B	0.046 (3)	0.039 (3)	0.048 (3)	0.009 (2)	−0.004 (2)	−0.005 (2)
N2B	0.029 (2)	0.033 (2)	0.044 (2)	0.0066 (18)	−0.0050 (18)	0.002 (2)
C1B	0.043 (3)	0.031 (3)	0.051 (3)	0.007 (2)	−0.004 (2)	0.006 (2)
C2B	0.035 (3)	0.042 (3)	0.033 (3)	0.000 (2)	0.008 (2)	−0.004 (2)
C3B	0.048 (3)	0.064 (4)	0.037 (3)	0.005 (3)	−0.006 (3)	0.005 (3)
C4B	0.048 (4)	0.104 (7)	0.029 (3)	−0.009 (4)	−0.003 (3)	−0.006 (4)
C5B	0.066 (4)	0.075 (5)	0.038 (3)	−0.024 (4)	0.000 (3)	−0.017 (3)
C6B	0.055 (3)	0.044 (3)	0.042 (3)	−0.017 (3)	0.006 (3)	−0.009 (3)
C7B	0.035 (3)	0.034 (3)	0.033 (3)	−0.006 (2)	0.004 (2)	−0.001 (2)
C8B	0.042 (3)	0.033 (3)	0.036 (3)	0.001 (2)	0.003 (2)	0.002 (2)
C9B	0.040 (3)	0.032 (3)	0.043 (3)	0.001 (2)	0.001 (2)	−0.002 (2)
C10B	0.035 (3)	0.028 (2)	0.039 (3)	−0.003 (2)	0.004 (2)	0.001 (2)
C11B	0.037 (3)	0.041 (3)	0.044 (3)	0.002 (2)	0.001 (2)	−0.002 (2)
C12B	0.022 (2)	0.039 (3)	0.031 (2)	−0.0030 (18)	0.0011 (17)	0.002 (2)
C13B	0.031 (2)	0.044 (3)	0.045 (3)	0.002 (2)	0.010 (2)	0.009 (3)
C14B	0.048 (3)	0.061 (4)	0.050 (4)	0.002 (3)	0.015 (3)	0.026 (3)
C15B	0.044 (4)	0.064 (5)	0.074 (5)	0.002 (3)	−0.015 (3)	0.017 (4)
C16B	0.045 (3)	0.042 (3)	0.066 (4)	0.017 (3)	0.008 (3)	0.016 (3)
S1C	0.0636 (9)	0.0291 (7)	0.0477 (8)	0.0017 (6)	0.0190 (7)	0.0010 (6)
N1C	0.059 (3)	0.042 (3)	0.052 (3)	−0.017 (3)	0.015 (2)	−0.007 (2)
N2C	0.037 (2)	0.028 (2)	0.053 (3)	−0.0032 (18)	0.019 (2)	−0.002 (2)
C1C	0.065 (4)	0.035 (3)	0.053 (3)	−0.013 (3)	0.024 (3)	0.000 (3)
C2C	0.043 (3)	0.045 (3)	0.036 (3)	−0.005 (2)	0.008 (2)	0.000 (2)
C3C	0.048 (4)	0.090 (6)	0.043 (3)	−0.017 (4)	0.010 (3)	0.009 (4)
C4C	0.056 (4)	0.112 (7)	0.031 (3)	−0.015 (4)	0.016 (3)	−0.008 (4)
C5C	0.061 (4)	0.081 (6)	0.053 (4)	0.004 (4)	0.017 (3)	−0.019 (4)
C6C	0.054 (4)	0.055 (4)	0.046 (3)	0.000 (3)	0.005 (3)	−0.009 (3)
C7C	0.036 (3)	0.045 (3)	0.032 (3)	−0.009 (2)	0.001 (2)	0.002 (2)
C8C	0.037 (3)	0.038 (3)	0.040 (3)	−0.005 (2)	0.010 (2)	−0.001 (2)
C9C	0.045 (3)	0.031 (3)	0.048 (3)	0.000 (2)	0.013 (2)	0.000 (2)
C10C	0.034 (3)	0.029 (3)	0.048 (3)	−0.0045 (19)	0.013 (2)	−0.008 (2)
C11C	0.048 (3)	0.038 (3)	0.041 (3)	−0.004 (2)	0.019 (2)	−0.003 (2)
C12C	0.029 (2)	0.038 (3)	0.035 (3)	−0.003 (2)	0.0045 (19)	0.002 (2)
C13C	0.042 (3)	0.043 (3)	0.044 (3)	0.000 (2)	0.014 (2)	0.010 (3)
C14C	0.066 (4)	0.053 (4)	0.070 (5)	−0.015 (3)	0.029 (4)	0.006 (4)
C15C	0.046 (3)	0.053 (4)	0.071 (5)	0.001 (3)	0.008 (3)	0.023 (4)
C16C	0.071 (5)	0.082 (6)	0.051 (4)	0.006 (4)	0.033 (4)	0.011 (4)
S1D	0.0509 (8)	0.0453 (8)	0.0521 (9)	−0.0110 (7)	0.0211 (7)	−0.0024 (7)
N1D	0.046 (2)	0.024 (2)	0.043 (2)	0.0009 (18)	0.016 (2)	−0.0039 (18)
N2D	0.042 (2)	0.039 (3)	0.040 (2)	0.0067 (19)	0.018 (2)	−0.002 (2)
C1D	0.062 (4)	0.033 (3)	0.044 (3)	0.002 (2)	0.021 (3)	−0.004 (2)
C2D	0.034 (2)	0.032 (3)	0.039 (3)	0.001 (2)	0.006 (2)	−0.001 (2)
C3D	0.054 (3)	0.047 (3)	0.044 (3)	0.002 (3)	0.011 (3)	−0.007 (3)
C4D	0.070 (5)	0.076 (5)	0.040 (3)	−0.012 (4)	0.026 (3)	−0.002 (3)
C5D	0.052 (4)	0.082 (5)	0.048 (4)	−0.014 (4)	0.015 (3)	0.020 (4)
C6D	0.044 (3)	0.036 (3)	0.045 (3)	−0.004 (2)	0.007 (2)	0.006 (2)
C7D	0.030 (2)	0.037 (3)	0.032 (2)	−0.0058 (19)	0.0069 (19)	0.001 (2)
C8D	0.038 (3)	0.027 (2)	0.030 (2)	−0.0027 (19)	0.006 (2)	−0.0019 (19)
C9D	0.028 (2)	0.033 (2)	0.031 (2)	0.0029 (18)	0.0077 (18)	0.0021 (19)
C10D	0.033 (2)	0.037 (3)	0.028 (2)	−0.0022 (19)	0.0070 (19)	0.001 (2)
C11D	0.033 (3)	0.068 (5)	0.052 (4)	−0.013 (3)	0.012 (2)	−0.002 (3)
C12D	0.032 (2)	0.050 (3)	0.037 (3)	0.002 (2)	0.010 (2)	−0.006 (2)
C13D	0.039 (3)	0.060 (4)	0.037 (3)	0.010 (3)	0.013 (2)	−0.007 (3)
C14D	0.221 (15)	0.068 (6)	0.070 (6)	0.094 (9)	0.000 (7)	−0.006 (5)
C15D	0.061 (4)	0.059 (4)	0.070 (5)	0.007 (3)	0.002 (4)	−0.028 (4)
C16D	0.084 (6)	0.106 (8)	0.111 (8)	−0.029 (6)	0.064 (6)	−0.045 (7)

### Geometric parameters (Å, º)

**Table:**

S1A—C11A	1.731 (6)	S1C—C11C	1.715 (6)
S1A—C10A	1.745 (5)	S1C—C10C	1.722 (6)
N1A—C1A	1.470 (7)	N1C—C9C	1.433 (8)
N1A—C9A	1.476 (6)	N1C—C1C	1.434 (8)
N1A—H1A	0.97 (2)	N1C—H1C	0.96 (2)
N2A—C10A	1.312 (6)	N2C—C10C	1.301 (8)
N2A—C12A	1.376 (7)	N2C—C12C	1.377 (7)
C1A—C2A	1.507 (9)	C1C—C2C	1.515 (9)
C1A—H1A1	0.9900	C1C—H1C1	0.9900
C1A—H1A2	0.9900	C1C—H1C2	0.9900
C2A—C7A	1.405 (9)	C2C—C7C	1.394 (8)
C2A—C3A	1.418 (8)	C2C—C3C	1.404 (9)
C3A—C4A	1.366 (12)	C3C—C4C	1.376 (13)
C3A—H3A	0.9500	C3C—H3C	0.9500
C4A—C5A	1.369 (13)	C4C—C5C	1.409 (14)
C4A—H4A	0.9500	C4C—H4C	0.9500
C5A—C6A	1.394 (9)	C5C—C6C	1.366 (10)
C5A—H5A	0.9500	C5C—H5C	0.9500
C6A—C7A	1.411 (8)	C6C—C7C	1.400 (9)
C6A—H6A	0.9500	C6C—H6C	0.9500
C7A—C8A	1.508 (7)	C7C—C8C	1.522 (8)
C8A—C9A	1.526 (7)	C8C—C9C	1.506 (8)
C8A—H8A1	0.9900	C8C—H8C1	0.9900
C8A—H8A2	0.9900	C8C—H8C2	0.9900
C9A—C10A	1.495 (7)	C9C—C10C	1.500 (8)
C9A—H9A	1.0000	C9C—H9C	1.0000
C11A—C12A	1.364 (9)	C11C—C12C	1.349 (8)
C11A—H11A	0.9500	C11C—H11C	0.9500
C12A—C13A	1.500 (8)	C12C—C13C	1.527 (8)
C13A—C16A	1.521 (9)	C13C—C14C	1.501 (10)
C13A—C15A	1.531 (9)	C13C—C15C	1.527 (9)
C13A—C14A	1.562 (10)	C13C—C16C	1.540 (9)
C14A—H14A	0.9800	C14C—H14G	0.9800
C14A—H14B	0.9800	C14C—H14H	0.9800
C14A—H14C	0.9800	C14C—H14I	0.9800
C15A—H15A	0.9800	C15C—H15G	0.9800
C15A—H15B	0.9800	C15C—H15H	0.9800
C15A—H15C	0.9800	C15C—H15I	0.9800
C16A—H16A	0.9800	C16C—H16G	0.9800
C16A—H16B	0.9800	C16C—H16H	0.9800
C16A—H16C	0.9800	C16C—H16I	0.9800
S1B—C11B	1.688 (6)	S1D—C11D	1.726 (7)
S1B—C10B	1.723 (5)	S1D—C10D	1.729 (6)
N1B—C1B	1.457 (7)	N1D—C9D	1.451 (7)
N1B—C9B	1.460 (7)	N1D—C1D	1.454 (7)
N1B—H1B	0.99 (2)	N1D—H1D	0.97 (2)
N2B—C10B	1.288 (7)	N2D—C10D	1.287 (7)
N2B—C12B	1.410 (6)	N2D—C12D	1.392 (7)
C1B—C2B	1.489 (8)	C1D—C2D	1.523 (8)
C1B—H1B1	0.9900	C1D—H1D1	0.9900
C1B—H1B2	0.9900	C1D—H1D2	0.9900
C2B—C7B	1.395 (8)	C2D—C7D	1.382 (8)
C2B—C3B	1.408 (8)	C2D—C3D	1.387 (8)
C3B—C4B	1.404 (12)	C3D—C4D	1.414 (11)
C3B—H3B	0.9500	C3D—H3D	0.9500
C4B—C5B	1.358 (13)	C4D—C5D	1.344 (13)
C4B—H4B	0.9500	C4D—H4D	0.9500
C5B—C6B	1.391 (10)	C5D—C6D	1.392 (9)
C5B—H5B	0.9500	C5D—H5D	0.9500
C6B—C7B	1.409 (8)	C6D—C7D	1.398 (8)
C6B—H6B	0.9500	C6D—H6D	0.9500
C7B—C8B	1.500 (7)	C7D—C8D	1.507 (7)
C8B—C9B	1.522 (8)	C8D—C9D	1.544 (7)
C8B—H8B1	0.9900	C8D—H8D1	0.9900
C8B—H8B2	0.9900	C8D—H8D2	0.9900
C9B—C10B	1.505 (7)	C9D—C10D	1.501 (7)
C9B—H9B	1.0000	C9D—H9D	1.0000
C11B—C12B	1.358 (8)	C11D—C12D	1.366 (10)
C11B—H11B	0.9500	C11D—H11D	0.9500
C12B—C13B	1.519 (8)	C12D—C13D	1.493 (8)
C13B—C16B	1.533 (9)	C13D—C14D	1.480 (11)
C13B—C14B	1.536 (8)	C13D—C16D	1.520 (10)
C13B—C15B	1.544 (9)	C13D—C15D	1.521 (10)
C14B—H14D	0.9800	C14D—H14J	0.9800
C14B—H14E	0.9800	C14D—H14K	0.9800
C14B—H14F	0.9800	C14D—H14L	0.9800
C15B—H15D	0.9800	C15D—H15J	0.9800
C15B—H15E	0.9800	C15D—H15K	0.9800
C15B—H15F	0.9800	C15D—H15L	0.9800
C16B—H16D	0.9800	C16D—H16J	0.9800
C16B—H16E	0.9800	C16D—H16K	0.9800
C16B—H16F	0.9800	C16D—H16L	0.9800
			
C11A—S1A—C10A	89.4 (3)	C11C—S1C—C10C	89.4 (3)
C1A—N1A—C9A	109.7 (4)	C9C—N1C—C1C	110.7 (5)
C1A—N1A—H1A	108 (4)	C9C—N1C—H1C	104 (6)
C9A—N1A—H1A	109 (5)	C1C—N1C—H1C	136 (5)
C10A—N2A—C12A	112.5 (4)	C10C—N2C—C12C	112.3 (5)
N1A—C1A—C2A	114.1 (5)	N1C—C1C—C2C	115.1 (5)
N1A—C1A—H1A1	108.7	N1C—C1C—H1C1	108.5
C2A—C1A—H1A1	108.7	C2C—C1C—H1C1	108.5
N1A—C1A—H1A2	108.7	N1C—C1C—H1C2	108.5
C2A—C1A—H1A2	108.7	C2C—C1C—H1C2	108.5
H1A1—C1A—H1A2	107.6	H1C1—C1C—H1C2	107.5
C7A—C2A—C3A	117.8 (6)	C7C—C2C—C3C	119.9 (7)
C7A—C2A—C1A	122.0 (5)	C7C—C2C—C1C	119.2 (5)
C3A—C2A—C1A	120.1 (6)	C3C—C2C—C1C	120.9 (6)
C4A—C3A—C2A	120.7 (7)	C4C—C3C—C2C	119.8 (8)
C4A—C3A—H3A	119.6	C4C—C3C—H3C	120.1
C2A—C3A—H3A	119.6	C2C—C3C—H3C	120.1
C3A—C4A—C5A	121.7 (7)	C3C—C4C—C5C	120.2 (6)
C3A—C4A—H4A	119.1	C3C—C4C—H4C	119.9
C5A—C4A—H4A	119.1	C5C—C4C—H4C	119.9
C4A—C5A—C6A	119.7 (7)	C6C—C5C—C4C	120.0 (8)
C4A—C5A—H5A	120.2	C6C—C5C—H5C	120.0
C6A—C5A—H5A	120.2	C4C—C5C—H5C	120.0
C5A—C6A—C7A	119.7 (7)	C5C—C6C—C7C	120.7 (7)
C5A—C6A—H6A	120.1	C5C—C6C—H6C	119.7
C7A—C6A—H6A	120.1	C7C—C6C—H6C	119.7
C2A—C7A—C6A	120.3 (5)	C2C—C7C—C6C	119.4 (6)
C2A—C7A—C8A	120.6 (5)	C2C—C7C—C8C	120.7 (6)
C6A—C7A—C8A	119.0 (5)	C6C—C7C—C8C	119.9 (6)
C7A—C8A—C9A	109.7 (4)	C9C—C8C—C7C	112.3 (5)
C7A—C8A—H8A1	109.7	C9C—C8C—H8C1	109.1
C9A—C8A—H8A1	109.7	C7C—C8C—H8C1	109.1
C7A—C8A—H8A2	109.7	C9C—C8C—H8C2	109.1
C9A—C8A—H8A2	109.7	C7C—C8C—H8C2	109.1
H8A1—C8A—H8A2	108.2	H8C1—C8C—H8C2	107.9
N1A—C9A—C10A	109.2 (4)	N1C—C9C—C10C	110.1 (5)
N1A—C9A—C8A	112.9 (4)	N1C—C9C—C8C	113.9 (5)
C10A—C9A—C8A	111.4 (4)	C10C—C9C—C8C	112.5 (5)
N1A—C9A—H9A	107.7	N1C—C9C—H9C	106.6
C10A—C9A—H9A	107.7	C10C—C9C—H9C	106.6
C8A—C9A—H9A	107.7	C8C—C9C—H9C	106.6
N2A—C10A—C9A	125.5 (4)	N2C—C10C—C9C	126.8 (5)
N2A—C10A—S1A	113.2 (4)	N2C—C10C—S1C	113.5 (4)
C9A—C10A—S1A	121.2 (4)	C9C—C10C—S1C	119.6 (4)
C12A—C11A—S1A	110.3 (4)	C12C—C11C—S1C	110.8 (4)
C12A—C11A—H11A	124.9	C12C—C11C—H11C	124.6
S1A—C11A—H11A	124.9	S1C—C11C—H11C	124.6
C11A—C12A—N2A	114.6 (5)	C11C—C12C—N2C	113.9 (5)
C11A—C12A—C13A	127.3 (5)	C11C—C12C—C13C	126.0 (5)
N2A—C12A—C13A	118.1 (5)	N2C—C12C—C13C	120.1 (5)
C12A—C13A—C16A	111.1 (6)	C14C—C13C—C12C	108.8 (5)
C12A—C13A—C15A	107.6 (5)	C14C—C13C—C15C	111.3 (6)
C16A—C13A—C15A	110.2 (6)	C12C—C13C—C15C	106.9 (5)
C12A—C13A—C14A	109.5 (5)	C14C—C13C—C16C	109.5 (6)
C16A—C13A—C14A	110.3 (6)	C12C—C13C—C16C	110.9 (6)
C15A—C13A—C14A	108.1 (6)	C15C—C13C—C16C	109.3 (6)
C13A—C14A—H14A	109.5	C13C—C14C—H14G	109.5
C13A—C14A—H14B	109.5	C13C—C14C—H14H	109.5
H14A—C14A—H14B	109.5	H14G—C14C—H14H	109.5
C13A—C14A—H14C	109.5	C13C—C14C—H14I	109.5
H14A—C14A—H14C	109.5	H14G—C14C—H14I	109.5
H14B—C14A—H14C	109.5	H14H—C14C—H14I	109.5
C13A—C15A—H15A	109.5	C13C—C15C—H15G	109.5
C13A—C15A—H15B	109.5	C13C—C15C—H15H	109.5
H15A—C15A—H15B	109.5	H15G—C15C—H15H	109.5
C13A—C15A—H15C	109.5	C13C—C15C—H15I	109.5
H15A—C15A—H15C	109.5	H15G—C15C—H15I	109.5
H15B—C15A—H15C	109.5	H15H—C15C—H15I	109.5
C13A—C16A—H16A	109.5	C13C—C16C—H16G	109.5
C13A—C16A—H16B	109.5	C13C—C16C—H16H	109.5
H16A—C16A—H16B	109.5	H16G—C16C—H16H	109.5
C13A—C16A—H16C	109.5	C13C—C16C—H16I	109.5
H16A—C16A—H16C	109.5	H16G—C16C—H16I	109.5
H16B—C16A—H16C	109.5	H16H—C16C—H16I	109.5
C11B—S1B—C10B	89.2 (3)	C11D—S1D—C10D	89.1 (3)
C1B—N1B—C9B	111.2 (5)	C9D—N1D—C1D	109.9 (4)
C1B—N1B—H1B	112 (5)	C9D—N1D—H1D	122 (5)
C9B—N1B—H1B	107 (5)	C1D—N1D—H1D	104 (4)
C10B—N2B—C12B	110.4 (5)	C10D—N2D—C12D	111.1 (5)
N1B—C1B—C2B	114.9 (5)	N1D—C1D—C2D	115.0 (5)
N1B—C1B—H1B1	108.5	N1D—C1D—H1D1	108.5
C2B—C1B—H1B1	108.5	C2D—C1D—H1D1	108.5
N1B—C1B—H1B2	108.5	N1D—C1D—H1D2	108.5
C2B—C1B—H1B2	108.5	C2D—C1D—H1D2	108.5
H1B1—C1B—H1B2	107.5	H1D1—C1D—H1D2	107.5
C7B—C2B—C3B	118.7 (6)	C7D—C2D—C3D	119.6 (5)
C7B—C2B—C1B	121.1 (5)	C7D—C2D—C1D	120.5 (5)
C3B—C2B—C1B	120.2 (6)	C3D—C2D—C1D	120.0 (5)
C4B—C3B—C2B	120.5 (7)	C2D—C3D—C4D	120.9 (6)
C4B—C3B—H3B	119.7	C2D—C3D—H3D	119.6
C2B—C3B—H3B	119.7	C4D—C3D—H3D	119.6
C5B—C4B—C3B	119.4 (6)	C5D—C4D—C3D	119.0 (6)
C5B—C4B—H4B	120.3	C5D—C4D—H4D	120.5
C3B—C4B—H4B	120.3	C3D—C4D—H4D	120.5
C4B—C5B—C6B	122.1 (7)	C4D—C5D—C6D	120.9 (7)
C4B—C5B—H5B	118.9	C4D—C5D—H5D	119.5
C6B—C5B—H5B	118.9	C6D—C5D—H5D	119.5
C5B—C6B—C7B	118.7 (7)	C5D—C6D—C7D	120.6 (6)
C5B—C6B—H6B	120.6	C5D—C6D—H6D	119.7
C7B—C6B—H6B	120.6	C7D—C6D—H6D	119.7
C2B—C7B—C6B	120.5 (5)	C2D—C7D—C6D	119.0 (5)
C2B—C7B—C8B	120.9 (5)	C2D—C7D—C8D	121.3 (5)
C6B—C7B—C8B	118.6 (5)	C6D—C7D—C8D	119.6 (5)
C7B—C8B—C9B	112.4 (5)	C7D—C8D—C9D	109.8 (4)
C7B—C8B—H8B1	109.1	C7D—C8D—H8D1	109.7
C9B—C8B—H8B1	109.1	C9D—C8D—H8D1	109.7
C7B—C8B—H8B2	109.1	C7D—C8D—H8D2	109.7
C9B—C8B—H8B2	109.1	C9D—C8D—H8D2	109.7
H8B1—C8B—H8B2	107.8	H8D1—C8D—H8D2	108.2
N1B—C9B—C10B	110.5 (5)	N1D—C9D—C10D	108.8 (4)
N1B—C9B—C8B	113.5 (5)	N1D—C9D—C8D	111.3 (4)
C10B—C9B—C8B	112.0 (5)	C10D—C9D—C8D	112.7 (4)
N1B—C9B—H9B	106.8	N1D—C9D—H9D	108.0
C10B—C9B—H9B	106.8	C10D—C9D—H9D	108.0
C8B—C9B—H9B	106.8	C8D—C9D—H9D	108.0
N2B—C10B—C9B	124.5 (5)	N2D—C10D—C9D	124.9 (5)
N2B—C10B—S1B	115.3 (4)	N2D—C10D—S1D	115.2 (4)
C9B—C10B—S1B	120.1 (4)	C9D—C10D—S1D	119.8 (4)
C12B—C11B—S1B	111.8 (4)	C12D—C11D—S1D	109.9 (5)
C12B—C11B—H11B	124.1	C12D—C11D—H11D	125.1
S1B—C11B—H11B	124.1	S1D—C11D—H11D	125.1
C11B—C12B—N2B	113.2 (5)	C11D—C12D—N2D	114.6 (5)
C11B—C12B—C13B	128.8 (5)	C11D—C12D—C13D	125.4 (6)
N2B—C12B—C13B	117.9 (5)	N2D—C12D—C13D	119.9 (5)
C12B—C13B—C16B	109.8 (5)	C14D—C13D—C12D	110.0 (6)
C12B—C13B—C14B	108.3 (5)	C14D—C13D—C16D	108.1 (9)
C16B—C13B—C14B	108.7 (6)	C12D—C13D—C16D	112.6 (7)
C12B—C13B—C15B	110.0 (5)	C14D—C13D—C15D	111.9 (9)
C16B—C13B—C15B	109.5 (6)	C12D—C13D—C15D	109.1 (5)
C14B—C13B—C15B	110.5 (6)	C16D—C13D—C15D	105.2 (6)
C13B—C14B—H14D	109.5	C13D—C14D—H14J	109.5
C13B—C14B—H14E	109.5	C13D—C14D—H14K	109.5
H14D—C14B—H14E	109.5	H14J—C14D—H14K	109.5
C13B—C14B—H14F	109.5	C13D—C14D—H14L	109.5
H14D—C14B—H14F	109.5	H14J—C14D—H14L	109.5
H14E—C14B—H14F	109.5	H14K—C14D—H14L	109.5
C13B—C15B—H15D	109.5	C13D—C15D—H15J	109.5
C13B—C15B—H15E	109.5	C13D—C15D—H15K	109.5
H15D—C15B—H15E	109.5	H15J—C15D—H15K	109.5
C13B—C15B—H15F	109.5	C13D—C15D—H15L	109.5
H15D—C15B—H15F	109.5	H15J—C15D—H15L	109.5
H15E—C15B—H15F	109.5	H15K—C15D—H15L	109.5
C13B—C16B—H16D	109.5	C13D—C16D—H16J	109.5
C13B—C16B—H16E	109.5	C13D—C16D—H16K	109.5
H16D—C16B—H16E	109.5	H16J—C16D—H16K	109.5
C13B—C16B—H16F	109.5	C13D—C16D—H16L	109.5
H16D—C16B—H16F	109.5	H16J—C16D—H16L	109.5
H16E—C16B—H16F	109.5	H16K—C16D—H16L	109.5
			
C9A—N1A—C1A—C2A	−44.2 (7)	C9C—N1C—C1C—C2C	−49.9 (8)
N1A—C1A—C2A—C7A	13.9 (9)	N1C—C1C—C2C—C7C	20.6 (9)
N1A—C1A—C2A—C3A	−169.5 (6)	N1C—C1C—C2C—C3C	−159.2 (6)
C7A—C2A—C3A—C4A	−1.3 (10)	C7C—C2C—C3C—C4C	0.3 (10)
C1A—C2A—C3A—C4A	−178.1 (7)	C1C—C2C—C3C—C4C	−179.9 (7)
C2A—C3A—C4A—C5A	−0.3 (13)	C2C—C3C—C4C—C5C	−0.1 (11)
C3A—C4A—C5A—C6A	1.5 (13)	C3C—C4C—C5C—C6C	0.6 (12)
C4A—C5A—C6A—C7A	−1.2 (11)	C4C—C5C—C6C—C7C	−1.3 (11)
C3A—C2A—C7A—C6A	1.5 (9)	C3C—C2C—C7C—C6C	−0.9 (9)
C1A—C2A—C7A—C6A	178.3 (6)	C1C—C2C—C7C—C6C	179.2 (6)
C3A—C2A—C7A—C8A	−178.0 (5)	C3C—C2C—C7C—C8C	178.0 (6)
C1A—C2A—C7A—C8A	−1.3 (8)	C1C—C2C—C7C—C8C	−1.8 (9)
C5A—C6A—C7A—C2A	−0.3 (9)	C5C—C6C—C7C—C2C	1.5 (10)
C5A—C6A—C7A—C8A	179.2 (5)	C5C—C6C—C7C—C8C	−177.5 (6)
C2A—C7A—C8A—C9A	19.0 (7)	C2C—C7C—C8C—C9C	12.1 (8)
C6A—C7A—C8A—C9A	−160.6 (5)	C6C—C7C—C8C—C9C	−169.0 (6)
C1A—N1A—C9A—C10A	−170.1 (5)	C1C—N1C—C9C—C10C	−170.5 (6)
C1A—N1A—C9A—C8A	65.3 (6)	C1C—N1C—C9C—C8C	62.1 (8)
C7A—C8A—C9A—N1A	−51.2 (6)	C7C—C8C—C9C—N1C	−42.2 (7)
C7A—C8A—C9A—C10A	−174.5 (4)	C7C—C8C—C9C—C10C	−168.4 (5)
C12A—N2A—C10A—C9A	−175.5 (5)	C12C—N2C—C10C—C9C	−176.4 (5)
C12A—N2A—C10A—S1A	1.8 (6)	C12C—N2C—C10C—S1C	0.7 (6)
N1A—C9A—C10A—N2A	−162.6 (5)	N1C—C9C—C10C—N2C	−132.1 (6)
C8A—C9A—C10A—N2A	−37.2 (7)	C8C—C9C—C10C—N2C	−3.8 (9)
N1A—C9A—C10A—S1A	20.4 (6)	N1C—C9C—C10C—S1C	51.0 (7)
C8A—C9A—C10A—S1A	145.7 (4)	C8C—C9C—C10C—S1C	179.2 (4)
C11A—S1A—C10A—N2A	−0.6 (4)	C11C—S1C—C10C—N2C	−1.6 (5)
C11A—S1A—C10A—C9A	176.8 (4)	C11C—S1C—C10C—C9C	175.7 (5)
C10A—S1A—C11A—C12A	−0.7 (5)	C10C—S1C—C11C—C12C	2.1 (5)
S1A—C11A—C12A—N2A	1.8 (7)	S1C—C11C—C12C—N2C	−2.2 (7)
S1A—C11A—C12A—C13A	−176.1 (5)	S1C—C11C—C12C—C13C	177.0 (4)
C10A—N2A—C12A—C11A	−2.4 (7)	C10C—N2C—C12C—C11C	1.0 (7)
C10A—N2A—C12A—C13A	175.8 (5)	C10C—N2C—C12C—C13C	−178.2 (5)
C11A—C12A—C13A—C16A	−1.2 (9)	C11C—C12C—C13C—C14C	129.0 (7)
N2A—C12A—C13A—C16A	−179.1 (6)	N2C—C12C—C13C—C14C	−51.9 (7)
C11A—C12A—C13A—C15A	119.5 (7)	C11C—C12C—C13C—C15C	−110.6 (7)
N2A—C12A—C13A—C15A	−58.4 (7)	N2C—C12C—C13C—C15C	68.5 (7)
C11A—C12A—C13A—C14A	−123.3 (7)	C11C—C12C—C13C—C16C	8.5 (9)
N2A—C12A—C13A—C14A	58.8 (7)	N2C—C12C—C13C—C16C	−172.4 (6)
C9B—N1B—C1B—C2B	−46.4 (8)	C9D—N1D—C1D—C2D	−44.6 (7)
N1B—C1B—C2B—C7B	17.8 (8)	N1D—C1D—C2D—C7D	11.4 (8)
N1B—C1B—C2B—C3B	−161.9 (6)	N1D—C1D—C2D—C3D	−168.6 (6)
C7B—C2B—C3B—C4B	−0.9 (9)	C7D—C2D—C3D—C4D	−1.0 (10)
C1B—C2B—C3B—C4B	178.8 (6)	C1D—C2D—C3D—C4D	179.0 (6)
C2B—C3B—C4B—C5B	1.1 (11)	C2D—C3D—C4D—C5D	−0.6 (12)
C3B—C4B—C5B—C6B	0.0 (12)	C3D—C4D—C5D—C6D	0.8 (12)
C4B—C5B—C6B—C7B	−1.4 (11)	C4D—C5D—C6D—C7D	0.6 (11)
C3B—C2B—C7B—C6B	−0.4 (8)	C3D—C2D—C7D—C6D	2.3 (8)
C1B—C2B—C7B—C6B	179.9 (6)	C1D—C2D—C7D—C6D	−177.7 (5)
C3B—C2B—C7B—C8B	178.6 (5)	C3D—C2D—C7D—C8D	−178.5 (5)
C1B—C2B—C7B—C8B	−1.2 (8)	C1D—C2D—C7D—C8D	1.5 (8)
C5B—C6B—C7B—C2B	1.5 (9)	C5D—C6D—C7D—C2D	−2.1 (9)
C5B—C6B—C7B—C8B	−177.5 (6)	C5D—C6D—C7D—C8D	178.7 (6)
C2B—C7B—C8B—C9B	13.0 (8)	C2D—C7D—C8D—C9D	17.9 (7)
C6B—C7B—C8B—C9B	−168.0 (5)	C6D—C7D—C8D—C9D	−163.0 (5)
C1B—N1B—C9B—C10B	−173.3 (5)	C1D—N1D—C9D—C10D	−169.1 (4)
C1B—N1B—C9B—C8B	59.8 (7)	C1D—N1D—C9D—C8D	66.2 (6)
C7B—C8B—C9B—N1B	−42.3 (7)	C7D—C8D—C9D—N1D	−51.7 (6)
C7B—C8B—C9B—C10B	−168.3 (5)	C7D—C8D—C9D—C10D	−174.3 (4)
C12B—N2B—C10B—C9B	−174.8 (5)	C12D—N2D—C10D—C9D	−176.7 (5)
C12B—N2B—C10B—S1B	2.9 (6)	C12D—N2D—C10D—S1D	0.5 (6)
N1B—C9B—C10B—N2B	−134.5 (6)	N1D—C9D—C10D—N2D	−163.7 (5)
C8B—C9B—C10B—N2B	−6.8 (8)	C8D—C9D—C10D—N2D	−39.7 (7)
N1B—C9B—C10B—S1B	48.0 (6)	N1D—C9D—C10D—S1D	19.3 (6)
C8B—C9B—C10B—S1B	175.6 (4)	C8D—C9D—C10D—S1D	143.2 (4)
C11B—S1B—C10B—N2B	−2.6 (5)	C11D—S1D—C10D—N2D	−0.2 (5)
C11B—S1B—C10B—C9B	175.1 (5)	C11D—S1D—C10D—C9D	177.1 (4)
C10B—S1B—C11B—C12B	1.5 (5)	C10D—S1D—C11D—C12D	−0.1 (5)
S1B—C11B—C12B—N2B	−0.3 (7)	S1D—C11D—C12D—N2D	0.4 (7)
S1B—C11B—C12B—C13B	176.0 (4)	S1D—C11D—C12D—C13D	−176.5 (5)
C10B—N2B—C12B—C11B	−1.6 (7)	C10D—N2D—C12D—C11D	−0.6 (7)
C10B—N2B—C12B—C13B	−178.4 (5)	C10D—N2D—C12D—C13D	176.6 (5)
C11B—C12B—C13B—C16B	129.8 (6)	C11D—C12D—C13D—C14D	−126.0 (10)
N2B—C12B—C13B—C16B	−54.0 (6)	N2D—C12D—C13D—C14D	57.2 (10)
C11B—C12B—C13B—C14B	−111.6 (7)	C11D—C12D—C13D—C16D	−5.4 (10)
N2B—C12B—C13B—C14B	64.5 (7)	N2D—C12D—C13D—C16D	177.8 (7)
C11B—C12B—C13B—C15B	9.2 (9)	C11D—C12D—C13D—C15D	110.9 (8)
N2B—C12B—C13B—C15B	−174.6 (6)	N2D—C12D—C13D—C15D	−65.9 (8)
